# Fibroblast Growth Factor 23 and Left Ventricular Hypertrophy in Chronic Kidney Disease—A Pediatric Perspective

**DOI:** 10.3389/fped.2021.702719

**Published:** 2021-08-04

**Authors:** Andrea Grund, Manish D. Sinha, Dieter Haffner, Maren Leifheit-Nestler

**Affiliations:** ^1^Department of Paediatric Kidney, Liver and Metabolic Diseases, Hannover Medical School Children's Hospital, Hanover, Germany; ^2^Paediatric Research Centre, Hannover Medical School, Hanover, Germany; ^3^Department of Paediatric Nephrology, King's College London, Evelina London Children's Hospital, London, United Kingdom

**Keywords:** fibroblast growth factor 23, left ventricular hypertrophy, children, chronic kidney disease - mineral and bone disease, chronic kidney disease

## Abstract

Cardiovascular diseases (CVD) are a hallmark in pediatric patients with chronic kidney disease (CKD) contributing to an enhanced risk of all-cause and CV morbidity and mortality in these patients. The bone-derived phosphaturic hormone fibroblast growth factor (FGF) 23 progressively rises with declining kidney function to maintain phosphate homeostasis, with up to 1,000-fold increase in patients with kidney failure requiring dialysis. FGF23 is associated with the development of left ventricular hypertrophy (LVH) and thereby accounts to be a CVD risk factor in CKD. Experimentally, FGF23 directly induces hypertrophic growth of cardiac myocytes *in vitro* and LVH *in vivo*. Further, clinical studies in adult CKD have observed cardiotoxicity associated with FGF23. Data regarding prevalence and determinants of FGF23 excess in children with CKD are limited. This review summarizes current data and discusses whether FGF23 may be a key driver of LVH in pediatric CKD.

## Introduction

Chronic kidney disease (CKD) is a significant global health issue and defined as gradual loss of kidney function ultimately leading to irreversible kidney damage (1). CKD patients present a high risk for cardiovascular events. Fifty percent which remain the predominant cause of patients with CKD stage 4 and 5 develop cardiovascular diseases (CVD), which cause of death with 40% mortality rate (2). The cardiorenal syndrome (CRS) type 4 reflects the complex relationship between primary CKD and resultant CVD (3, 4). Left ventricular hypertrophy (LVH), cardiac fibrosis and vascular diseases remain the most common cardiac abnormalities in CRS 4, with 90% of adult patients with kidney failure developing LVH (5, 6). The primary causes of cardiac-associated death in these adults on haemodialysis are stroke, congestive heart failure, myocardial infarction and cardiac arrest (7, 8). Childhood CKD is also associated with amplified cardiovascular (CV) morbidity and mortality both during childhood with progressive worsening in those with childhood-onset CKD become young adults (7–9). The severity of CVDs in CKD patients is aggravated by the mineral bone disorder of CKD, called Chronic Kidney Disease-Mineral and Bone Disorder (CKD-MBD). Thus, CKD-MBD is a CKD-specific cardiovascular risk factor and displays changes in mineral metabolism parameters including phosphate, calcium, parathyroid hormone (PTH), calcitriol (1,25-dihydroxyvitamin D3, or 1,25D), and fibroblast growth factor 23 (FGF23) (10). In children with CKD impaired bone architecture may result in bone pain and deformities, short stature, fractures and ectopic calcifications. In children, LVH is the commonest CV abnormality with reported prevalence between 17 and 55%, increasing prevalence with worsening GFR (11–14). As LVH and its progression are associated with adverse CV outcomes in patients with CKD (15), it remains important to understand additional risk factors. The present narrative review focusses on FGF23, a bone-derived phosphaturic hormone regulating renal phosphate homeostasis, in the development of LVH in pediatric CKD and summarizes recent clinical literature in this field.

## General Mechanisms of CVD in CKD

Mainly, there are two different processes involved in the development of CVD. As a response to mechanical or hemodynamic overload, a remodeling process leads to hypertrophy of the left ventricle (LV) (16). There is evidence, that LV dysfunction in children with CKD predisposes them to LVH (17). Pressure overload resulting from prolonged hypertension leads to concentric hypertrophy, whereas eccentric hypertrophy is a cause of volume overload due to e.g., aortic and mitral valve insufficiency (18). These two different mechanisms induce distinct patterns of cardiac myocyte contractile protein (sarcomere) formation. New sarcomeres are added in parallel with relative increase in the width of the myocytes during pressure-induced concentric LVH. The pronounced increase in wall thickness with only little expansion of the LV cavity is a direct effect of the myocyte growth. During eccentric hypertrophy the LV cavity together with wall thickness is increased. Here, sarcomeres are added in series producing longitudinal growth of cardiac myocytes. Importantly, when myocytes elongated only in length without an increase in diameter, the LV dilates. Both, pressure and volume overload trigger the activation of multiple signaling pathways leading to myocardial remodeling (18). In CKD patients, these mechanisms can be activated independently, since here numerous humoral factors are altered *a priori*. To make matters worse, different hemodynamic stimuli e.g., hypertension, arterial stiffness, volume expansion, and anemia can occur simultaneously in CKD, resulting in distinct alterations of LV geometry (19). Hypertrophy of the LV becomes maladaptive with decreased capillary density, arrhythmia, and myocardial fibrosis leading to myocyte death causing diastolic and systolic dysfunction. The activation of the renin–angiotensin–aldosterone system (RAAS) is also known to induce LVH (20, 21). FGF23 can stimulate RAAS directly by inhibiting renal Angiotensin Converting Enzyme 2 (ACE2), thus inhibiting its vasodilatory and hypotensive properties resulting in promotion of vasoconstrictive and inflammatory effects of angiotensin II (22–24). Vice versa, FGF23 expression may also be stimulated by RAAS activation (25). Interestingly, FGF23 blunts the protective effects of angiotensin receptor blocker (ARBs) on the kidney and mitigates the expression of anti-inflammatory genes in experimental renal failure (26). Since, hypertension is one of the most common comorbidities in CKD patients, FGF23-mediated activation of RAAS may play an important role in hypertension in CKD.

Vascular injury is promoting CVD as well. Changes in vascular tissue, leading to atherosclerotic and arteriosclerotic alterations and/or vascular calcification, are very common in CKD. Plaque and atheroma formation during atherosclerosis start with accumulation of macrophages in the vascular intima followed by aggregation of lipids, different fibers of collagen and smooth muscle cells. At the same time, calcification with the development of atherosclerotic lesions at the intima occurs causing stenosis and local closures of the artery. Circulating endothelial progenitor cells (EPCs) contribute to angiogenetic processes where they are recruited to the heart vessels after injury or damage. Studies show a reduced number of EPCs with impaired function in CVD (27).

In arteriosclerosis, the process of arterial stiffening and arterial calcification results in arteries with thicker intima and media, which affects their elasticity. The increased wall thickness together with a lumen enlargement, resulting in higher systolic blood pressure and arterial stiffening, characterize the vascular remodeling processes during arteriosclerosis. The main consequences of arterial stiffening are LVH and an altered coronary perfusion (28).

Over the past decade, vascular calcification in combination with chronic inflammation have come into focus as another risk factor for CVD in CKD, driving disease progression particularly in children on chronic dialysis (29). In combination with oxidative stress, inflammation is implicated in the progression of CKD due to increased production of pro-inflammatory cytokines such as C-reactive protein (CRP), interleukin-6 (IL-6), interleukin-1 (IL-1) and tumor necrosis factor-α (TNF-α) with IL6 and TNF-α being strong inducers of vascular calcification (30). In the Modification of Diet in Renal Disease (MDRD) study and in the Trial to Reduce Cardiovascular Events With Aranesp Therapy (TREAT) higher CRP levels have been independently linked to CVD prevalence in diabetic and non-diabetic kidney disease (31, 32). Mechanistically, CRP has been shown to enhance transdifferentiation of vascular smooth muscle cells (VSMC) via an Fc fragment of IgG receptor IIa (FCGR2A)-dependent activation of oxidative stress (33). Loss of VSMCs quiescent phenotype and their transdifferentiation into osteoblastic-like cells with increased mineralized matrix secretion lead to the progression of vascular calcification (34).

The pro-inflammatory marker IL-6 is involved in the development of atherosclerotic lesions by recruiting inflammatory cells. Circulating plasma level of IL-6 were elevated in a cohort of stable haemodialysis patients enrolled in the HEMO study suggesting IL-6 to be a strong predictor of CV and all-cause mortality (35, 36). Furthermore, in a recent clinical study measuring IL-6 in patients with coronary artery calcification in CKD, IL-6 was found to be a strong and independent predictor of CV and all-cause death (37).

TNF-α is induced by inflammatory stimuli and stimulates the immune response. It was shown to be upregulated in kidney disease and LVH in patients receiving dialysis (38). Increased level of circulating TNF-α are observed in myocardial dysfunction and fibrosis and it is thought to be involved in the development of atherosclerosis by impairing endothelial function (39). Despite these data, currently there is no therapy available for the treatment of inflammation to decrease CV calcification in those with CKD. Recently, using an experimental CKD model, Singh et al. reported that activation of FGFR4 and PLCg/calcineurin/NFAT signaling by FGF23 in the liver stimulated an inflammatory response (40). Blockade of FGF receptor 4 (FGFR4) resulted in reduced circulating CRP levels. Since FGFR4 is expressed in a wide range of tissue, this FGFR isoform might be a promising target for clinical intervention.

## CVD in Pediatric CKD

In children, LVH develops early in mild and moderate chronic renal insufficiency and exacerbates with kidney failure requiring dialysis (9, 11, 41). Children with CKD show the same risk factors that predict the development of cardiac hypertrophy as adults, although the duration of the “abnormality” is shorter. Importantly however, in contradistinction to adults, children do not have long-standing hypertension and other major co-morbidities including diabetes and smoking. Amongst modifiable risk associations for LVH in children, anemia, increasing ponderosity, hypertension and treatment with antihypertensive medications that do not target RAAS e.g., vasodilators, have been reported previously (42–44). Numerous studies have observed a correlation of increased PTH levels and the progression of LVH in children with CKD stages 2–4 with PTH having a direct effect on cardiac myocytes (45, 46). Thus, Bakkaloglu et al. show in the IPPN cohort for children with CKD an increased risk for developing LVH of over 70% (47).

Endothelial dysfunction (ED) measured by impaired endothelium-dependent flow-mediated dilation (FMD) is observed in children with advanced kidney failure, on chronic dialysis and after renal transplantation (48, 49). Nitric oxide deficiency and oxidative stress are discussed as cause and consequence of ED (50, 51). In addition, vascular abnormalities display a high prevalence in pediatric CKD. Medial vascular calcification due to its association with increased vascular stiffening and cardiac workload, poor coronary perfusion, and sudden cardiac death, is responsible for the high risk for CV mortality even in young adults with CKD and this risk may be comparable to the older people in the general population (52, 53). The association between CKD and accelerated vascular aging is evident and discussed intensively, but so far little is known about the underlying molecular mechanisms (54–56). A dysregulated calcium (Ca) and phosphate (P) metabolism accelerate vascular calcification in CKD that is promoted by the death of vascular smooth muscle cells and osteogenic differentiation (57, 58). Major physiological regulators of Ca and P metabolism are FGF23 and its co-receptor α-Klotho as well as PTH, 25-hydroxyvitamin D (25OHD) and calcitriol (59). Recently, a focus on uremic toxins has been evaluated to account for vascular calcification and fibrosis. Indoxyl sulfate (IS), a metabolite of the tryptophan metabolism that accumulates in the plasma during kidney failure, for example correlates with congestive heart failure in patients with CKD (60). IS induces the expression of runt-related transcription factor 2 (Runx2) and osteopontin (OPN) and activates the PI3K/Akt/NF-κB pathway, all of them participating in the differentiation of vascular smooth muscle cells (VSMCs) from a contractile to osteogenic phenotype thereby promoting vascular calcification in CKD (61–63). Furthermore, IS has pro-hypertrophic, pro-fibrotic, and pro-inflammatory properties and stimulates hypertrophy in neonatal rat cardiomyocytes and collagen synthesis in neonatal rat cardiac fibroblasts via activation of MAP kinases and NF-κB pathways (64).

## FGF23 in CKD

**Figure 1:**
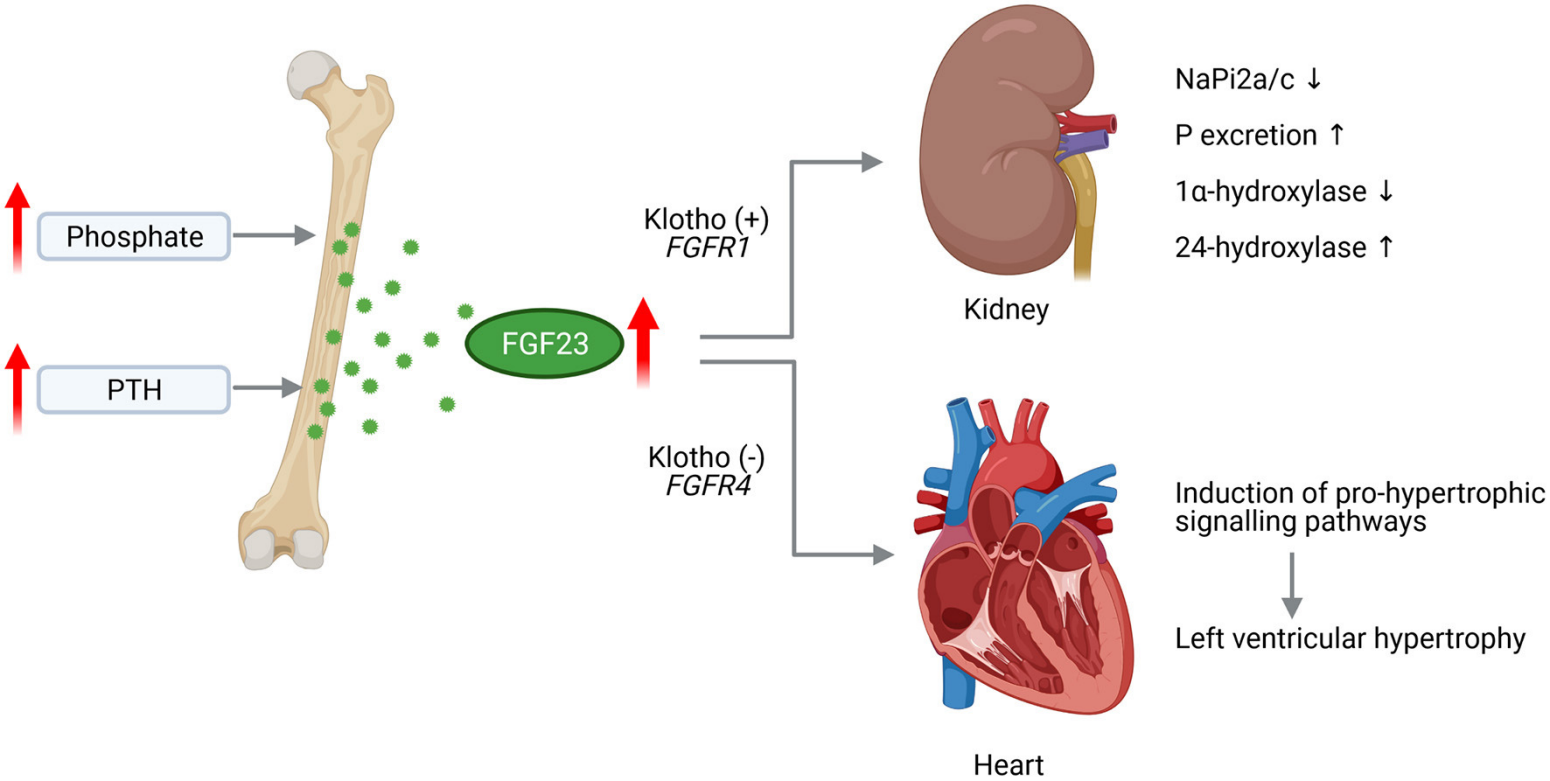
Pathological effects of FGF23 in CKD on the kidney and heart. Chronically high phosphate load and hypersecretion of parathyroid hormone (PTH) stimulate the synthesis of fibroblast growth factor (FGF) 23 in the bone. Increased level of circulating FGF23 activates FGF receptor (FGFR) 1/Klotho complex in the kidney leading to decreased expression of sodium phosphate transporters NaPi2a and NaPi2c enhancing phosphate (P) excretion. FGF23 further supresses 1a-hydroxylase and increases 24-hydroxylase resulting in reduced serum 1,25D level. Acting on the heart, FGF23 activates FGFR4 independent of Klotho that induces pro-hypertrophic signaling pathways promoting the development of left ventricular hypertrophy (This figure is created with BioRender.com).

FGF23 physiologically binds to the FGF receptors (FGFR) 1/α-Klotho complex in the kidney to mediate downregulation of sodium-dependent phosphate co-transporters (NaPi2a and NaPi2c) that results in decreased tubular phosphate reabsorption and finally lower serum phosphate levels (Figure 1). In early stages of CKD, the increased level of FGF23 go along with unchanged level of phosphate and PTH (65, 66). The advanced progression of CKD results in phosphate retention that increases FGF23 levels further on (67, 68). At the same time, PTH levels rise and 1,25D serum concentrations decrease to diminish phosphate absorption further on (68). The decline of 1,25D due to elevated FGF23 is prolonged via suppression of renal 1α-hydroxylase, the enzyme that catalyzes the synthesis of 1,25D from the major circulating metabolite 25OHD, as well as induction of 24-hydroxylase, the enzyme responsible for production of 24,25(OH)_2_D_3_ (69). Thus, reduction of intestinal calcium absorption due to decreased 1,25D levels in conjunction with low ionized calcium leading to pronounced increase of PTH level and ultimately in secondary hyperparathyroidism.

Thus, alterations to levels of FGF23, which progressively increase together with changes in the metabolism of phosphate, calcium, PTH and/or 1,25D in worsening renal dysfunction with CKD result in CKD-MBD. This complex clinical syndrome is characterized by abnormalities in bone and mineral metabolism as well as extra-skeletal calcification resulting in fractures and bone deformities and, most prominently, in poor growth (10, 70), which all of them contribute to enhanced risk of CVD, fractures and mortality across all stages of CKD. The pathogenesis of CKD-MBD is complex, involving regulatory mechanism in bone, kidney, heart and parathyroid glands.

## FGF23 and LVH in CKD

In early CKD, circulating FGF23 levels rise exponentially and are up-to 1,000-fold enhanced in kidney failure (65, 66). Elevated FGF23 levels are associated with all-cause and CV mortality in the general population (71, 72) as well as in CKD patients (73), LV dysfunction and atrial fibrillation (AF) in patients without CV co-morbidities (74, 75), and the development of cardiac hypertrophy (76) and fibrosis (77). Thereby, cardiac remodeling was shown to be stimulated by activation of myocardial FGFR4 although independent of α-Klotho (Figure 1) (78, 79). Higher FGF23 levels were further observed in stable ischaemic cardiomyopathy (72) and heart failure (HF) with reduced ejection fraction (HFrEF) (80–82).

The role of FGF23 in the development of LVH in children with CKD was investigated intensively since the last decade. Thereby it was shown that FGF23 level are markedly increased in haemodialysis patients with prevalent concentric LVH (66, 83), while in a study cohort of 83 children with non-dialysis stages 3–5 CKD no significant relationship between circulating FGF23 and LVH was observed (84). However, the Chronic Kidney Disease in Children (CKiD) study enrolled children with mild-to-moderate pre-dialysis CKD, respectively, concluded that a high plasma FGF23 concentration above 170 RU/ml together with estimated GFR ≥ 45 ml/min per 1.73 m^2^ is associated with a higher prevalence of LVH (85, 86). Importantly, these studies differ in methods and number of enrolled children. Sinha et al. measured serum intact FGF23 in 83 children, whereas Portale et al. and Mitsnefes et al. measured C-terminal FGF23 plasma level in 419 and 587 children, respectively. This is controversial, since on the one hand intact FGF23 level are varying with a diurnal rhythm and have a high intraindividual variation (87). Furthermore, carefully handling is needed when processing K2-EDTA plasma, otherwise intact FGF23 will be unstable (88). On the other hand, intact FGF23 may reflect the biological relevance more than C-terminal FGF23 does (89). There is evidence, that the active full-length form is counter-regulated by the C-terminal fragments (90). Also, it was shown that the effect of dietary phosphate restriction on FGF23 has a greater impact on intact FGF23 than on the C-terminal form (91). The situation is complicated further by the fact that the four commercially available FGF23 assays are not validated for clinical use and are not standardized (92, 93).

**Figure 2:**
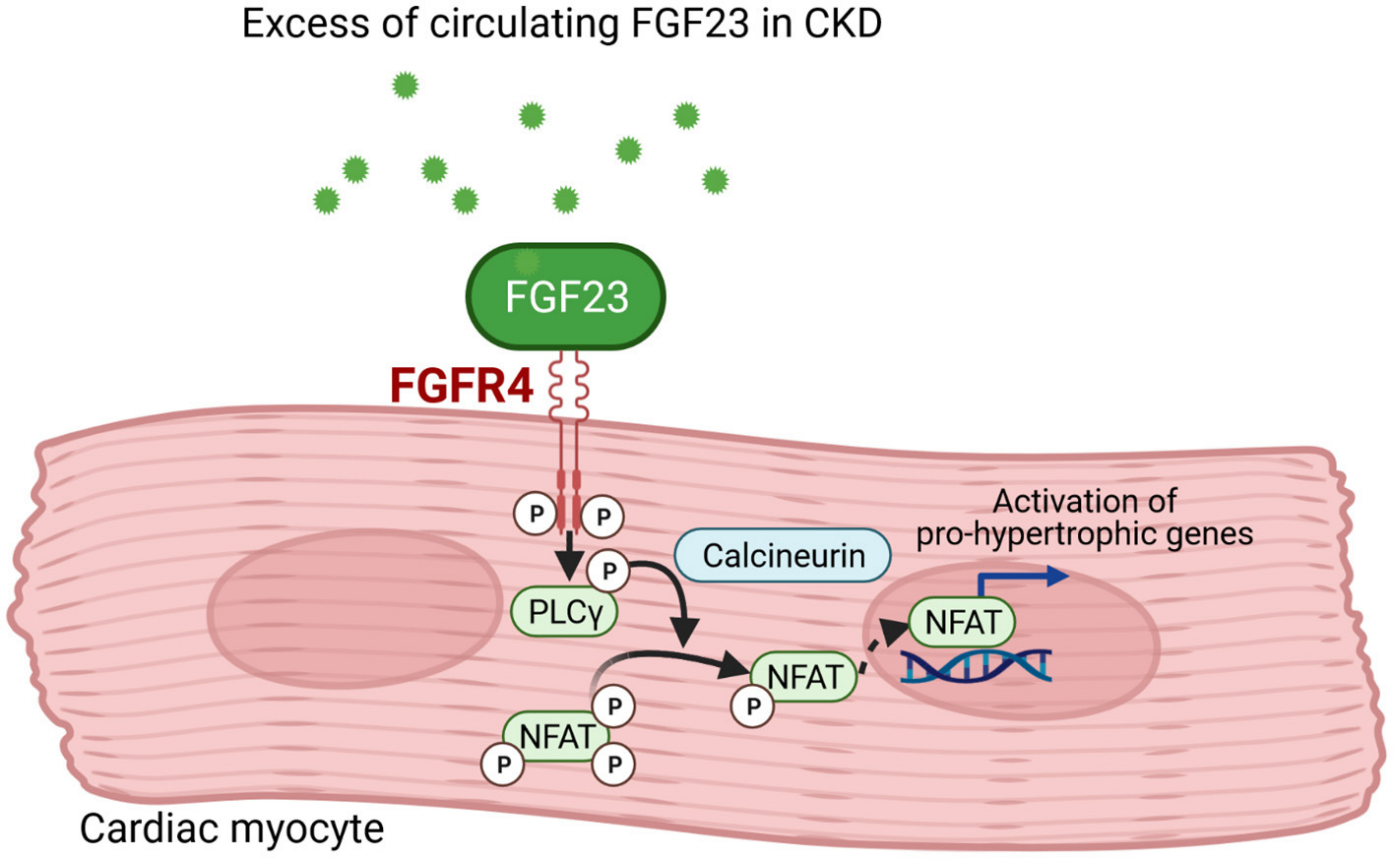
FGF23 signaling in cardiac myocytes. Fibroblast growth factor (FGF) 23 binds to FGF receptor (FGFR) 4 in cardiac myocytes that results in phosphorylation of phospholipase Cγ (PLCγ). The PLCγ-mediated activation of calcineurin dephosphorylates nuclear factor of activated T-cells (NFAT) that translocates into the nucleus and induces pro-hypertrophic target genes involved in cardiac remodeling (This figure is created with BioRender.com).

Mechanistically, it was shown that FGF23 directly induces hypertrophic growth of cardiac myocytes *in vitro* and cardiac hypertrophy *in vivo* that was abolished by inhibition of phospholipase Cγ (PLCγ) or calcineurin, but not by the use of MAP kinase, PI3 kinase or Akt inhibitors. Also, the use of PD173074 to block all FGFRs reduced LV mass and the cardiac expression of genes associated with LVH (94). A few years later, FGFR4 was identified to be the main effector of FGF23 signaling in the heart (78) leading to the conclusion that cardiac hypertrophy induced by FGF23 is mediated by the FGFR4/PLCγ/calcineurin pathway that activate nuclear factor of activated T-cells (NFAT) and consequently pro-hypertrophic NFAT target genes (Figure 2). A second mode of action for FGF23's pro-hypertrophic properties could be the direct regulation of the sodium-chloride channel NCC in distal renal tubules by FGF23 rendering it to be a sodium-conserving hormone (95). A connection of FGF23, cardiac hypertrophy and the local RAAS in the heart is also discussed. In 5/6 nephrectomised rats as experimental model of uraemia, an increase in size of myocyte as well as enhanced cardiac fibrosis together with induction of cardiac *Fgf23* and RAAS-associated gene expression was overserved (23). Paricalcitol mediated downregulation of RAAS and NFAT, respectively, together with diminished FGF23 level could thus attenuate cardiac hypertrophy (96, 97). In a retrospective case–control study in 24 pediatric CKD patients with kidney failure the relevance of FGF23 on cardiac fibrosis was evaluated (98). Increased collagen type I and III together with enhanced expression of pro-fibrotic growth factors and components of RAAS were observed, leading to the conclusion that FGF23 is induced by activated RAAS thereby promoting cardiac fibrosis. *In vitro*, FGF23-induced hypertrophic growth of isolated neonatal rat ventricular myocytes (NRVM) could be reversed by incubation with cyclosporine A, losartan and spironolactone (25). Furthermore, FGF23-mediated induction of TGF-β and CTGF in neonatal cardiac fibroblasts (NRCF) could be suppressed by losartan and spironolactone. Thus, leading to the conclusion that beside FGFR4/PLCγ/calcineurin signaling pathway, FGF23 promotes cardiac hypertrophy and fibrosis via activation of intra-cardiac RAAS.

During LVH, cardiac *Fgf23* expression is higher in cardiomyocyte-specific calcineurin A transgenic mice with normal *Fgf23* mRNA levels in bone (99). This phenomenon was also observed in an animal model as well as in humans with myocardial infarction (100, 101), indicating that an intra-cardiac synthesis of FGF23 may trigger a pathological cardiac phenotype in a paracrine manner. The latter is in line with a study in myocardial autopsy samples of pediatric patients with kidney failure showing in association of high cardiac FGF23 expression with LVH (79). Moreover, Leifheit-Nestler and Grabner et al. postulate in addition to FGF23 that the upregulation of FGFR4 in the myocardium of uremic rats further contributes to the development of LVH (102). Paradoxically, different mouse models of X-linked hypophosphatemia (XLH) as well as patients with XLH develop no cardiac hypertrophy although presenting high FGF23 level (103–105). The different clinical phenotype of XLH with its low serum phosphate and calcium level, normal renal function and no signs of atherosclerosis could be an explanation for those findings. Interestingly, a recent study in mice showed that a conditional deletion of Fgf23 in late osteoblast/osteocyte (*Fgf23*^*fl*/*fl*^*/Dmp1-Cre*^+^) followed by inducing CKD using an adenine-containing diet did not prevent hyperparathyroidism. Rather increased phosphate level as well as more pronounced cardiac hypertrophy were observed compared to uremic controls leading the authors to the conclusion that elevated FGF23 level in CKD may have a protective role to maintain cardiac homeostasis (106). Taken together, both the latter study and the studies in XLH conclude that phosphate may be a key mineral for FGF23 to exert its cardiotoxicity. However, further studies need to investigate the relationship between high FGF23 levels and altered phosphate homeostasis.

Cardiac hypertrophy is often accompanied by myocardial fibrosis, an excessive proliferation of fibroblast resulting in stiffening of cardiac tissue thereby giving resistance to pressure overload, but also stimulating diastolic dysfunction. FGF23 was recently discovered to be involved in the development of cardiac fibrosis. In a rat model of cardiac hypertrophy by pulmonary artery banding, FGF23 expression was highly upregulated in cardiac myocytes. Myocytes then secreted FGF23 to promote the activation of fibroblasts together with TGF-β1 and to transform fibroblasts into myofibroblasts via FGFR1 (107). Finally, FGF23 levels correlated with fibrosis in heart failure patients with preserved ejection fraction (HFpEF) (108). However, the expression of cardiac FGF23 did not correlate with the amount of cardiac fibrosis in pediatric patients with kidney failure (95). Thus, the impact of FGF23 on the development of cardiac fibrosis has to be investigated in future studies in more detail.

## Therapeutic Approaches to Reduce FGF23's CV Comorbidities

**Figure 3:**
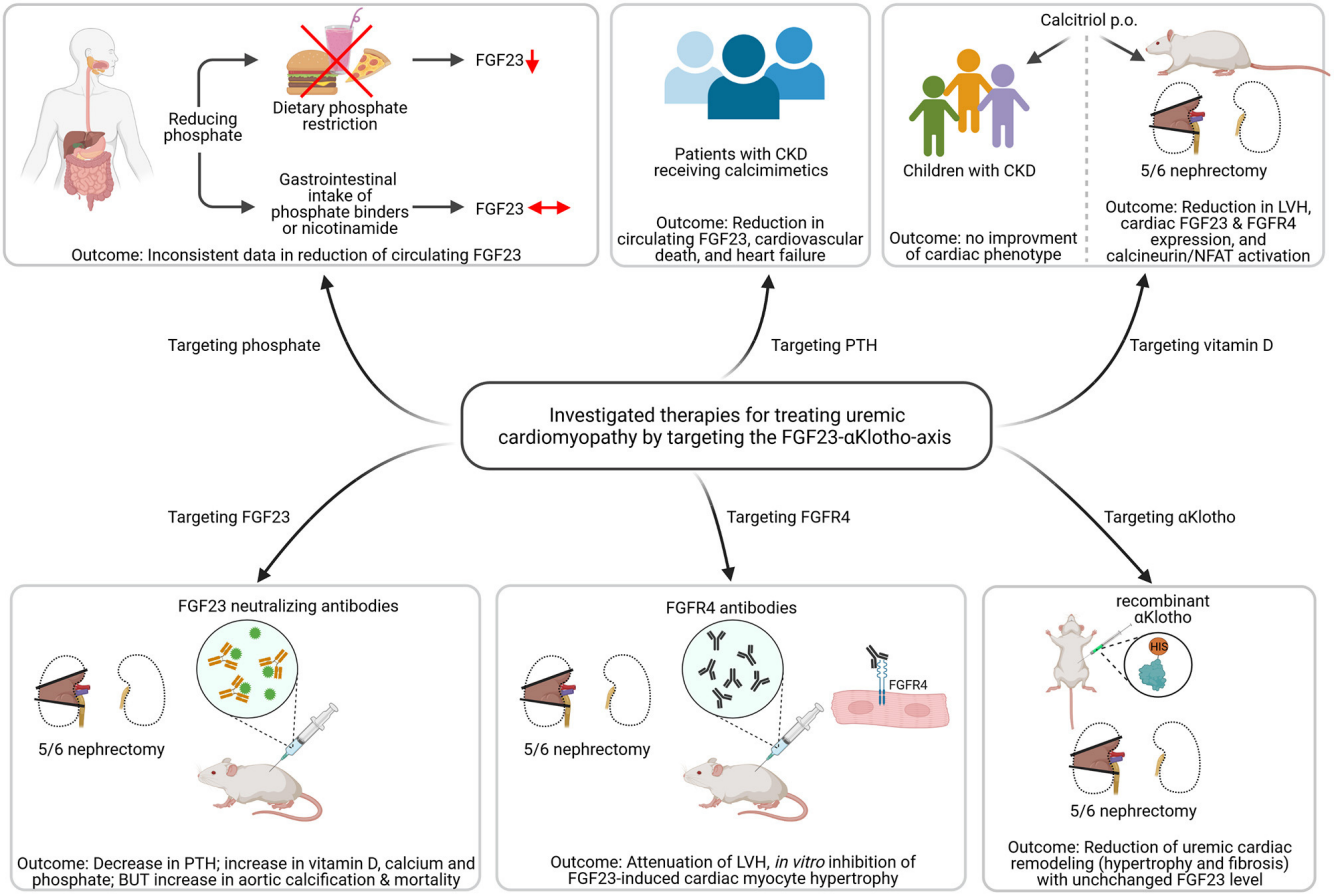
Investigated strategies targeting the FGF23-αKlotho-axis to ameliorate uremic cardiomyopathy. Dietary phosphate restriction causes a reduction in circulating levels of fibroblast growth factor (FGF) 23, while the use of phosphate binders or nicotinamide do not. Patients with chronic kidney disease (CKD) receiving calcimimetics show lower circulating FGF23 levels associated with reduced cardiovascular death and heart failure. Calcitriol therapy in 5/6 nephrectomized rats causes less activation of the FGF23/FGF receptor (FGFR) 4/calcineurin/nuclear factor of activated T-cells (NFAT) signaling pathway and improved left ventricular hypertrophy (LVH). However, treatment with active vitamin D shows no improvement of cardiac phenotype. The use of FGF23 neutralizing antibodies in uremic rats leads to decreased parathyroid hormone (PTH) levels and increased vitamin D, but causes aortic calcification and enhanced mortality. In contrast, targeting FGFR4 using anti-FGFR4 neutralizing antibodies attenuated LVH *in vivo* and inhibits FGF23-induced cardiac myocyte hypertrophy *in vitro*. Treatment with recombinant αKlotho reduces uremic cardiac remodeling without affecting high FGF23 levels (This figure is created with BioRender.com).

The aim of any therapeutic approach in pediatric CKD is to delay the progression of kidney failure and to improve CVD outcomes. FGF23 is associated with adverse CV outcome in CKD-MBD and it is thought to be a promising biomarker and a target for future therapeutic approaches. One of the most important studies that directly targeted FGF23 by using anti-FGF23 antibodies failed to improve the CV outcome in uremic rats and even increased mortality due to high phosphate-mediated vascular calcifications (Figure 3) (109). Whether therapeutic use of anti-FGF23 antibodies in dialysis patients, relieved of chronic phosphate load by dialysis, is more effective in reducing cardiac remodeling remains an open question.

Several studies target dietary phosphate restriction to lower FGF23 levels (Figure 3). Thereby, dietary phosphate restriction in healthy participants resulted in decreased intact FGF23 level, whereas phosphate-enriched diets increased intact FGF23 (110). The same findings were observed in studies of CKD and phosphate-restricted vs. phosphate-enriched diets (111, 112). However, it was further reported that C-terminal FGF23 was not changed regardless of the dietary phosphate content (113, 114). A recently published randomized clinical trial in stage 3b/4 CKD patients, the CKD Optimal Management with BInders and NicotinamidE (COMBINE) study, hypothesized that lowering phosphate level in early CKD prevents rising level of FGF23 and with that FGF23-associated morbidity and mortality (Figure 3). However, a 12 months therapy with lanthanum carbonate and/or nicotinamide failed to lower serum phosphate and FGF23 levels (115). The same was observed in other studies (116–118). Calcimimetics turned out to be more effective in lowering FGF23 and with that lowering mortality and CVD events significantly in patients with kidney failure (Figure 3) (119). However, further studies need to confirm the promising beneficial use of calcimimetics on cardiac function in the future and to elucidate the exact molecular mechanisms of its possible cardioprotection.

Further studies included the treatment of Vitamin D deficiency in 5/6 nephrectomized rats with calcitriol that reduced the expression of FGF23 and FGFR4 in the heart inactivating calcineurin/NFAT signaling and thereby reduced the development of LVH (102). However, active vitamin D treatment failed to improve LVH in pediatric CKD patients (Figure 3) (120). Targeting FGFR4 with neutralizing antibodies attenuated LVH in animal models of CKD or high phosphate load by reducing cardiac remodeling without affecting FGF23 level (78). However, clinical studies on the therapeutic benefit of anti-FGFR4 antibodies in LVH are still pending. Treatment of uremic cardiomyopathy in rodents with recombinant αKlotho ameliorated LVH and fibrosis independent of high FGF23 synthesis (Figure 3), but again, clinical studies are lacking to prove the positive effects of Klotho therapy on the CV system (121).

Currently, FGF23 concentrations are not routinely measured in patients. One reason is the absence of reliable immunoassays for measurement of FGF23, which are validated for clinical use (93, 122). Another reason is the reduced stability of biologically active intact FGF23 when blood processing is delayed (123), thus making it difficult to apply its use in routine clinical work. It remains questionable if FGF23 is a good predictor for progression of CKD. So far, only one study in adults with CKD has shown a benefit of adding FGF23 to existing prediction models for CKD progression (124), whereas all other studies have thus far failed to show any advantage in risk prediction for CKD onset when including FGF23 (125–127). Nevertheless, for the prediction of all-cause mortality in CKD patients, FGF23 seems to be a better prognostic marker (125, 128). Regarding CV outcomes, studies remain inconsistent. Alderson et al. point toward FGF23 to be a good predictor of CV events in pre-dialysis patients (128). Besides, Emrich et al. suggest that inclusion of NT-proBNP into the prediction model eliminates the association between FGF23 level and CV outcome (129). However, there is evidence that measured FGF23 levels can nevertheless predict the accessibility of patients to certain therapeutic approaches as shown for the treatment of patients with e.g., stable ischemic heart disease with angiotensin-converting enzyme inhibitor (130).

Experimental and clinical data reviewed here suggest it remains reasonable to consider that reducing elevated level of FGF23 is potentially a promising therapeutic pathway for reducing CVD in CKD in adult and children. Randomized clinical trials targeting the pathophysiology of FGF23-induced CVD are needed to help clarify the impact of FGF23 on LVH in CKD.

## Conclusion

In summary, the scientific community gained a lot of knowledge about the regulation and effects of FGF23. Although FGF23 mainly regulates phosphate homeostasis, in adults as well as in pediatric CKD the levels of FGF23 are markedly increased with progressing kidney failure and high FGF23 levels are strongly associated with left ventricular hypertrophy. However, a direct link to other CKD associated CV abnormalities needs to be proven in pediatric patients. In fact, CV events e.g., cardiac arrest and arrhythmia, are the leading cause of death in children with CKD and a dysregulated FGF23 metabolism may additionally have detrimental effects on the cardiovascular system. Regardless, many findings are contradictory and large prospective clinical studies are needed to elucidate the exact pathological mechanisms of FGF23 induced CVDs in pediatric CKD.

## Author Contributions

AG performed literature research, drafted the manuscript, and designed the figures. MS critically discussed content of review and all revisions of the manuscript. DH critically discussed the content of the review and revised the manuscript. ML-N critically discussed the content of the review and revised the manuscript and the figures. All authors contributed to the article and approved the submitted version.

## Conflict of Interest

The authors declare that the research was conducted in the absence of any commercial or financial relationships that could be construed as a potential conflict of interest.

## Publisher's Note

All claims expressed in this article are solely those of the authors and do not necessarily represent those of their affiliated organizations, or those of the publisher, the editors and the reviewers. Any product that may be evaluated in this article, or claim that may be made by its manufacturer, is not guaranteed or endorsed by the publisher.

## References

[1] Levey AS Coresh J Bolton K Culleton B Harvey KS Ikizler TA. K/DOQI clinical practice guidelines for chronic kidney disease: evaluation, classification, and stratification. Am J Kidney Dis. (2002) 39:S1–266. 11904577

[2] Thompson S James M Wiebe N Hemmelgarn B Manns B Klarenbach S. Cause of death in patients with reduced kidney function. JASN. (2015) 26:2504–11. 10.1681/ASN.2014070714 25733525 PMC4587695

[3] Clementi A Virzì GM Goh CY Cruz DN Granata A Vescovo G. Cardiorenal syndrome type 4: a review. Cardiorenal Med. (2013) 3:63–70. 10.1159/000350397 23946725 PMC3743409

[4] Granata A Clementi A Virzì GM Brocca A Cal M de Scarfia VR. Cardiorenal syndrome type 4: from chronic kidney disease to cardiovascular impairment. Eur J Intern Med. (2016) 30:1–6. 10.1016/j.ejim.2016.02.019 26961461

[5] Di Lullo L Bellasi A Barbera V Russo D Russo L Di Iorio B. Pathophysiology of the cardio-renal syndromes types 1-5: an uptodate. Indian Heart J. (2017) 69:255–65. 10.1016/j.ihj.2017.01.005 28460776 PMC5415026

[6] Himmelfarb J Ikizler TA. Hemodialysis. New Engl J Med. (2010) 363:1833–45. 10.1056/NEJMra0902710 21047227

[7] McDonald SP Craig JC. Long-term survival of children with end-stage renal disease. New Engl J Med. (2004) 350:2654–62. 10.1056/NEJMoa031643 15215481

[8] Groothoff JW Gruppen MP Offringa M Hutten J Lilien MR van de Kar NJ. Mortality and causes of death of end-stage renal disease in children: a Dutch cohort study. Kidney Int. (2002) 61:621–9. 10.1046/j.1523-1755.2002.00156.x 11849405

[9] Kouri AM Rheault MN. Cardiovascular disease in children with chronic kidney disease. Curr Opin Nephrol Hy. (2021) 30:231–6. 10.1097/MNH.0000000000000684 33464005

[10] Moe S Drüeke T Cunningham J Goodman W Martin K Olgaard K. Definition, evaluation, and classification of renal osteodystrophy: a position statement from Kidney Disease: Improving Global Outcomes (KDIGO). Kidney Int. (2006) 69:1945–53. 10.1038/sj.ki.5000414 16641930

[11] Matteucci MC Wühl E Picca S Mastrostefano A Rinelli G Romano C. Left ventricular geometry in children with mild to moderate chronic renal insufficiency. JASN. (2006) 17:218–26. 10.1681/ASN.2005030276 16280471

[12] Mitsnefes M Flynn J Cohn S Samuels J Blydt-Hansen T Saland J. Masked hypertension associates with left ventricular hypertrophy in children with CKD. JASN. (2010) 21:137–44. 10.1681/ASN.2009060609 19917781 PMC2799282

[13] Sinha MD Tibby SM Rasmussen P Rawlins D Turner C Dalton RN. Blood pressure control and left ventricular mass in children with chronic kidney disease. CJASN. (2011) 6:543–51. 10.2215/CJN.04690510 21115627 PMC3082412

[14] Doyon A Haas P Erdem S Ranchin B Kassai B Mencarelli F. Impaired systolic and diastolic left ventricular function in children with chronic kidney disease - results from the 4C study. Sci Rep. (2019) 9:11462. 10.1038/s41598-019-46653-3 31391470 PMC6685994

[15] Foley RN Parfrey PS Harnett JD Kent GM Murray DC Barré PE. The prognostic importance of left ventricular geometry in uremic cardiomyopathy. JASN. (1995) 5:2024–31. 10.1681/ASN.V5122024 7579050

[16] Heineke J Molkentin JD. Regulation of cardiac hypertrophy by intracellular signalling pathways. Nat Rev Mol Cell Bio. (2006) 7:589–600. 10.1038/nrm1983 16936699

[17] Gu H Sinha MD Li Y Simpson J Chowienczyk PJ. Elevated ejection-phase myocardial wall stress in children with chronic kidney disease. Hypertension. (2015) 66:823–9. 10.1161/HYPERTENSIONAHA.115.05704 26324503

[18] Pitoulis FG Terracciano CM. Heart plasticity in response to pressure- and volume-overload: a review of findings in compensated and decompensated phenotypes. Front Physiol. (2020) 11:92. 10.3389/fphys.2020.00092 32116796 PMC7031419

[19] Nitta K Iimuro S Imai E Matsuo S Makino H Akizawa T. Risk factors for increased left ventricular hypertrophy in patients with chronic kidney disease: findings from the CKD-JAC study. Clin Exp Nephrol. (2019) 23:85–98. 10.1007/s10157-018-1605-z 29951723 PMC6344393

[20] Li YC Kong J Wei M Chen Z-F Liu SQ Cao L-P. 1,25-Dihydroxyvitamin D(3) is a negative endocrine regulator of the renin-angiotensin system. J Clin Invest. (2002) 110:229–38. 10.1172/JCI0215219 12122115 PMC151055

[21] Xiang W Kong J Chen S Cao L-P Qiao G Zheng W. Cardiac hypertrophy in vitamin D receptor knockout mice: role of the systemic and cardiac renin-angiotensin systems. Am J Physiol Endocrinol Metab. (2005) 288:E125–32. 10.1152/ajpendo.00224.2004 15367398

[22] Leifheit-Nestler M Haffner D. Paracrine effects of FGF23 on the heart. Front Endocriniol. (2018) 9:278. 10.3389/fendo.2018.00278 PMC5985311 29892269

[23] Dai B David V Martin A Huang J Li H Jiao Y. A comparative transcriptome analysis identifying FGF23 regulated genes in the kidney of a mouse CKD model. PLoS ONE. (2012) 7:e44161. 10.1371/journal.pone.0044161 22970174 PMC3435395

[24] Prieto MC González-Villalobos RA Botros FT Martin VL Pagán J Satou R. Reciprocal changes in renal ACE/ANG II and ACE2/ANG 1-7 are associated with enhanced collecting duct renin in Goldblatt hypertensive rats. Am J Physiol Renal Physiol. (2011) 300:F749–55. 10.1152/ajprenal.00383.2009 21209009 PMC3064128

[25] Böckmann I Lischka J Richter B Deppe J Rahn A Fischer D-C. FGF23-mediated activation of local RAAS promotes cardiac hypertrophy and fibrosis. Int J Mol Sci. (2019) 20:4634. 10.3390/ijms20184634 PMC6770314 31540546

[26] Jong MA de Mirkovic K Mencke R Hoenderop JG Bindels RJ Vervloet MG. Fibroblast growth factor 23 modifies the pharmacological effects of angiotensin receptor blockade in experimental renal fibrosis. Nephrol Dial Transplant. (2017) 32:73–80. 10.1093/ndt/gfw105 27220755

[27] Shantsila E Watson T Lip GY. Endothelial progenitor cells in cardiovascular disorders. J Am Coll Cardiol. (2007) 49:741–52. 10.1016/j.jacc.2006.09.050 17306702

[28] London GM Marchais SJ Guérin AP Métivier F. Arteriosclerosis, vascular calcifications and cardiovascular disease in uremia. Curr Opin Nephrol Hy. (2005) 14:525–31. 10.1097/01.mnh.0000168336.67499.c0 16205470

[29] Goldstein SL Leung JC Silverstein DM. Pro- and anti-inflammatory cytokines in chronic pediatric dialysis patients: effect of aspirin. CJASN. (2006) 1:979–86. 10.2215/CJN.02291205 17699316

[30] Mihai S Codrici E Popescu ID Enciu A-M Albulescu L Necula LG. Inflammation-related mechanisms in chronic kidney disease prediction, progression, and outcome. J Immunol Res. (2018) 2018:2180373. 10.1155/2018/2180373 30271792 PMC6146775

[31] Menon V Wang X Greene T Beck GJ Kusek JW Marcovina SM. Relationship between C-reactive protein, albumin, and cardiovascular disease in patients with chronic kidney disease. Am J Kidney Dis. (2003) 42:44–52. 10.1016/S0272-6386(03)00407-4 12830455

[32] Mc Causland FR Claggett B Burdmann EA Eckardt K-U Kewalramani R Levey AS. C-reactive protein and risk of ESRD: results from the trial to reduce cardiovascular events with aranesp therapy (TREAT). Am J Kidney Dis. (2016) 68:873–81. 10.1053/j.ajkd.2016.07.022 27646425 PMC5123931

[33] Henze LA Luong TT Boehme B Masyout J Schneider MP Brachs S. Impact of C-reactive protein on osteo-/chondrogenic transdifferentiation and calcification of vascular smooth muscle cells. Aging. (2019) 11:5445–62. 10.18632/aging.102130 31377747 PMC6710049

[34] Leopold JA. Vascular calcification: mechanisms of vascular smooth muscle cell calcification. Trends Cardiovasc Med. (2015) 25:267–74. 10.1016/j.tcm.2014.10.021 25435520 PMC4414672

[35] Rao M Guo D Perianayagam MC Tighiouart H Jaber BL Pereira BJ. Plasma interleukin-6 predicts cardiovascular mortality in hemodialysis patients. Am J Kidney Dis. (2005) 45:324–33. 10.1053/j.ajkd.2004.09.018 15685511

[36] Hartman J Frishman WH. Inflammation and atherosclerosis: a review of the role of interleukin-6 in the development of atherosclerosis and the potential for targeted drug therapy. Cardiol Rev. (2014) 22:147–51. 10.1097/CRD.0000000000000021 24618929

[37] Kamińska J Stopiński M Mucha K Jedrzejczak A Gołebiowski M Niewczas MA. IL 6 but not TNF is linked to coronary artery calcification in patients with chronic kidney disease. Cytokine. (2019) 120:9–14. 10.1016/j.cyto.2019.04.002 30991230

[38] Gupta J Dominic EA Fink JC Ojo AO Barrows IR Reilly MP. Association between inflammation and cardiac geometry in chronic kidney disease: findings from the CRIC Study. PLoS ONE. (2015) 10:e0124772. 10.1371/journal.pone.0124772 25909952 PMC4409366

[39] Chia S Qadan M Newton R Ludlam CA Fox KA Newby DE. Intra-arterial tumor necrosis factor-alpha impairs endothelium-dependent vasodilatation and stimulates local tissue plasminogen activator release in humans. Arterioscler Thromb Vasc Biol. (2003) 23:695–701. 10.1161/01.ATV.0000065195.22904.FA 12692009

[40] Singh S Grabner A Yanucil C Schramm K Czaya B Krick S. Fibroblast growth factor 23 directly targets hepatocytes to promote inflammation in chronic kidney disease. Kidney Int. (2016) 90:985–96. 10.1016/j.kint.2016.05.019 27457912 PMC5065745

[41] Gruppen MP Groothoff JW Prins M van der Wouw P Offringa M Bos WJ. Cardiac disease in young adult patients with end-stage renal disease since childhood: a Dutch cohort study. Kidney Int. (2003) 63:1058–65. 10.1046/j.1523-1755.2003.00814.x 12631088

[42] Wilson AC Mitsnefes MM. Cardiovascular disease in CKD in children: update on risk factors, risk assessment, and management. Am J Kidney Dis. (2009) 54:345–60. 10.1053/j.ajkd.2009.04.027 19619845 PMC2714283

[43] Pugh D Gallacher PJ Dhaun N. Management of hypertension in chronic kidney disease. Drugs. (2019) 79:365–79. 10.1007/s40265-019-1064-1 30758803 PMC6422950

[44] Jackson RE Bellamy MC. Antihypertensive drugs. BJA Educ. (2015) 15:280–5. 10.1093/bjaceaccp/mku061

[45] Mitsnefes MM Kimball TR Kartal J Witt SA Glascock BJ Khoury PR. Progression of left ventricular hypertrophy in children with early chronic kidney disease: 2-year follow-up study. J Pediatr. (2006) 149:671–5. 10.1016/j.jpeds.2006.08.017 17095341

[46] Schlüter K-D Piper HM. Cardiovascular actions of parathyroid hormone and parathyroid hormone-related peptide. Cardiovasc Res. (1998) 37:34–41. 10.1016/S0008-6363(97)00194-6 9539855

[47] Bakkaloglu SA Borzych D Soo Ha I Serdaroglu E Büscher R Salas P. Cardiac geometry in children receiving chronic peritoneal dialysis: findings from the International Pediatric Peritoneal Dialysis Network (IPPN) registry. CJASN. (2011) 6:1926–33. 10.2215/CJN.05990710 21737855 PMC3359542

[48] Kari JA Donald AE Vallance DT Bruckdorfer KR Leone A Mullen MJ. Physiology and biochemistry of endothelial function in children with chronic renal failure. Kidney Int. (1997) 52:468–72. 10.1038/ki.1997.354 9264003

[49] Lilien MR Koomans HA Schröder CH. Hemodialysis acutely impairs endothelial function in children. Pediatr Nephrol. (2005) 20:200–4. 10.1007/s00467-004-1718-3 15627169

[50] Drozdz D Kwinta P Sztefko K Kordon Z Drozdz T Łatka M. Oxidative stress biomarkers and left ventricular hypertrophy in children with chronic kidney disease. Oxid Med Cell Longev. (2016) 2016:7520231. 10.1155/2016/7520231 26885251 PMC4739446

[51] Khandelwal P Murugan V Hari S Lakshmy R Sinha A Hari P. Dyslipidemia, carotid intima-media thickness and endothelial dysfunction in children with chronic kidney disease. Pediatr Nephrol. (2016) 31:1313–20. 10.1007/s00467-016-3350-4 26921213

[52] Goodman WG Goldin J Kuizon BD Yoon C Gales B Sider D. Coronary-artery calcification in young adults with end-stage renal disease who are undergoing dialysis. New Engl J Med. (2000) 342:1478–83. 10.1056/NEJM200005183422003 10816185

[53] Foley RN Parfrey PS Sarnak MJ. Clinical epidemiology of cardiovascular disease in chronic renal disease. Am J Kidney Dis. (1998) 32:S112–9. 10.1053/ajkd.1998.v32.pm9820470 9820470

[54] Kovacic JC Moreno P Hachinski V Nabel EG Fuster V. Cellular senescence, vascular disease, and aging: part 1 of a 2-part review. Circulation. (2011) 123:1650–60. 10.1161/CIRCULATIONAHA.110.007021 21502583

[55] Kovacic JC Moreno P Nabel EG Hachinski V Fuster V. Cellular senescence, vascular disease, and aging: part 2 of a 2-part review: clinical vascular disease in the elderly. Circulation. (2011) 123:1900–10. 10.1161/CIRCULATIONAHA.110.009118 21537006

[56] Sanchis P Ho CY Liu Y Beltran LE Ahmad S Jacob AP. Arterial “inflammaging” drives vascular calcification in children on dialysis. Kidney Int. (2019) 95:958–72. 10.1016/j.kint.2018.12.014 30827513 PMC6684370

[57] Shroff RC Donald AE Hiorns MP Watson A Feather S Milford D. Mineral metabolism and vascular damage in children on dialysis. JASN. (2007) 18:2996–3003. 10.1681/ASN.2006121397 17942964

[58] Shanahan CM Crouthamel MH Kapustin A Giachelli CM. Arterial calcification in chronic kidney disease: key roles for calcium and phosphate. Circ Res. (2011) 109:697–711. 10.1161/CIRCRESAHA.110.234914 21885837 PMC3249146

[59] Kuro-o M. Overview of the FGF23-Klotho axis. Pediatr Nephrol. (2010) 25:583–90. 10.1007/s00467-009-1260-4 19626341

[60] Hung S-C Kuo K-L Wu C-C Tarng D-C. Indoxyl sulfate: a novel cardiovascular risk factor in chronic kidney disease. J Am Heart Assoc. (2017) 6:e005022. 10.1161/JAHA.116.005022 28174171 PMC5523780

[61] Muteliefu G Enomoto A Jiang P Takahashi M Niwa T. Indoxyl sulphate induces oxidative stress and the expression of osteoblast-specific proteins in vascular smooth muscle cells. Nephrol Dial Transplant. (2009) 24:2051–8. 10.1093/ndt/gfn757 19164326

[62] Adijiang A Goto S Uramoto S Nishijima F Niwa T. Indoxyl sulphate promotes aortic calcification with expression of osteoblast-specific proteins in hypertensive rats. Nephrol Dial Transplant. (2008) 23:1892–901. 10.1093/ndt/gfm861 18334529

[63] He X Jiang H Gao F Liang S Wei M Chen L. Indoxyl sulfate-induced calcification of vascular smooth muscle cells via the PI3K/Akt/NF-κB signaling pathway. Microsc Res Tech. (2019) 82:2000–6. 10.1002/jemt.23369 31448474

[64] Lekawanvijit S Adrahtas A Kelly DJ Kompa AR Wang BH Krum H. Does indoxyl sulfate, a uraemic toxin, have direct effects on cardiac fibroblasts and myocytes? Eur Heart J. (2010) 31:1771–9. 10.1093/eurheartj/ehp574 20047993

[65] Isakova T Wahl P Vargas GS Gutiérrez OM Scialla J Xie H. Fibroblast growth factor 23 is elevated before parathyroid hormone and phosphate in chronic kidney disease. Kidney Int. (2011) 79:1370–8. 10.1038/ki.2011.47 21389978 PMC3134393

[66] Portale AA Wolf M Jüppner H Messinger S Kumar J Wesseling-Perry K. Disordered FGF23 and mineral metabolism in children with CKD. CJASN. (2014) 9:344–53. 10.2215/CJN.05840513 24311704 PMC3913243

[67] Saito H Maeda A Ohtomo S-I Hirata M Kusano K Kato S. Circulating FGF-23 is regulated by 1alpha,25-dihydroxyvitamin D3 and phosphorus *in vivo*. J Biol Chem. (2005) 280:2543–9. 10.1074/jbc.M408903200 15531762

[68] Portale AA Booth BE Halloran BP Morris RC. Effect of dietary phosphorus on circulating concentrations of 1,25-dihydroxyvitamin D and immunoreactive parathyroid hormone in children with moderate renal insufficiency. Eur J Clin Invest. (1984) 73:1580–9. 10.1172/JCI111365 6547151 PMC437069

[69] Shimada T Kakitani M Yamazaki Y Hasegawa H Takeuchi Y Fujita T. Targeted ablation of Fgf23 demonstrates an essential physiological role of FGF23 in phosphate and vitamin D metabolism. J Clin Invest. (2004) 113:561–8. 10.1172/JCI200419081 14966565 PMC338262

[70] Denburg MR Kumar J Jemielita T Brooks ER Skversky A Portale AA. Fracture burden and risk factors in childhood CKD: results from the CKiD Cohort Study. JASN. (2016) 27:543–50. 10.1681/ASN.2015020152 26139439 PMC4731126

[71] Ix JH Katz R Kestenbaum BR Boer IH de Chonchol M Mukamal KJ. Fibroblast growth factor-23 and death, heart failure, and cardiovascular events in community-living individuals: CHS (Cardiovascular Health Study). J Am Coll Cardiol. (2012) 60:200–7. 10.1016/j.jacc.2012.03.040 22703926 PMC3396791

[72] Lutsey PL Alonso A Selvin E Pankow JS Michos ED Agarwal SK. Fibroblast growth factor-23 and incident coronary heart disease, heart failure, and cardiovascular mortality: the Atherosclerosis Risk in Communities study. J Am Heart Assoc. (2014) 3:e000936. 10.1161/JAHA.114.000936 24922628 PMC4309096

[73] Mehta R Cai X Lee J Scialla JJ Bansal N Sondheimer JH. Association of fibroblast growth factor 23 with atrial fibrillation in chronic kidney disease, from the chronic renal insufficiency cohort study. JAMA Cardiology. (2016) 1:548–56. 10.1001/jamacardio.2016.1445 27434583 PMC4992989

[74] Seiler S Cremers B Rebling NM Hornof F Jeken J Kersting S. The phosphatonin fibroblast growth factor 23 links calcium-phosphate metabolism with left-ventricular dysfunction and atrial fibrillation. Eur Heart J. (2011) 32:2688–96. 10.1093/eurheartj/ehr215 21733911

[75] Chua W Purmah Y Cardoso VR Gkoutos GV Tull SP Neculau G. Data-driven discovery and validation of circulating blood-based biomarkers associated with prevalent atrial fibrillation. Eur Heart J. (2019) 40:1268–76. 10.1093/eurheartj/ehy815 30615112 PMC6475521

[76] Faul C Amaral AP Oskouei B Hu M-C Sloan A Isakova T. FGF23 induces left ventricular hypertrophy. JCI. (2011) 121:4393–408. 10.1172/JCI46122 21985788 PMC3204831

[77] Grabner A Schramm K Silswal N Hendrix M Yanucil C Czaya B. FGF23/FGFR4-mediated left ventricular hypertrophy is reversible. Sci Rep. (2017) 7:1993. 10.1038/s41598-017-02068-6 28512310 PMC5434018

[78] Grabner A Amaral AP Schramm K Singh S Sloan A Yanucil C. Activation of cardiac fibroblast growth factor receptor 4 causes left ventricular hypertrophy. Cell Metab. (2015) 22:1020–32. 10.1016/j.cmet.2015.09.002 26437603 PMC4670583

[79] Leifheit-Nestler M große Siemer R Flasbart K Richter B Kirchhoff F Ziegler WH. Induction of cardiac fgf23/fgfr4 expression is associated with left ventricular hypertrophy in patients with chronic kidney disease. Nephrol Dial Transplant. (2016) 31:1088–99. 10.1093/ndt/gfv421 26681731 PMC6388939

[80] koller l kleber me brandenburg vm goliasch g richter b sulzgruber p. fibroblast growth factor 23 is an independent and specific predictor of mortality in patients with heart failure and reduced ejection fraction. Circ Heart Fail. (2015) 8:1059–67. 10.1161/CIRCHEARTFAILURE.115.002341 26273098

[81] Plischke M Neuhold S Adlbrecht C Bielesz B Shayganfar S Bieglmayer C. Inorganic phosphate and FGF-23 predict outcome in stable systolic heart failure. Eur J Clin Invest. (2012) 42:649–56. 10.1111/j.1365-2362.2011.02631.x 22150123

[82] Jeinsen B von Sopova K Palapies L Leistner DM Fichtlscherer S Seeger FH. Bone marrow and plasma FGF-23 in heart failure patients: novel insights into the heart-bone axis. ESC Heart Fail. (2019) 6:536–44. 10.1002/ehf2.12416 30912310 PMC6487718

[83] Seeherunvong W Abitbol CL Chandar J Rusconi P Zilleruelo GE Freundlich M. Fibroblast growth factor 23 and left ventricular hypertrophy in children on dialysis. Pediatr Nephrol. (2012) 27:2129–36. 10.1007/s00467-012-2224-7 22710695

[84] Sinha MD Turner C Booth CJ Waller S Rasmussen P Goldsmith DJ. Relationship of FGF23 to indexed left ventricular mass in children with non-dialysis stages of chronic kidney disease. Pediatr Nephrol. (2015) 30:1843–52. 10.1007/s00467-015-3125-3 25975437

[85] Mitsnefes MM Betoko A Schneider MF Salusky IB Wolf MS Jüppner H. FGF23 and left ventricular hypertrophy in children with CKD. CJASN. (2018) 13:45–52. 10.2215/CJN.02110217 29025789 PMC5753303

[86] Portale AA Wolf MS Messinger S Perwad F Jüppner H Warady BA. Fibroblast growth factor 23 and risk of CKD progression in children. CJASN. (2016) 11:1989–98. 10.2215/CJN.02110216 27561289 PMC5108188

[87] Smith ER Cai MM McMahon LP Holt SG. Biological variability of plasma intact and C-terminal FGF23 measurements. J Clin Endocr Metab. (2012) 97:3357–65. 10.1210/jc.2012-1811 22689697

[88] Smith ER Ford ML Tomlinson LA Weaving G Rocks BF Rajkumar C. Instability of fibroblast growth factor-23 (FGF-23): implications for clinical studies. Clin Chim Acta. (2011) 412:1008–11. 10.1016/j.cca.2011.02.009 21324311

[89] Shimada T Muto T Urakawa I Yoneya T Yamazaki Y Okawa K. Mutant FGF-23 responsible for autosomal dominant hypophosphatemic rickets is resistant to proteolytic cleavage and causes hypophosphatemia *in vivo*. Endocrinology. (2002) 143:3179–82. 10.1210/endo.143.8.8795 12130585

[90] Goetz R Nakada Y Hu MC Kurosu H Wang L Nakatani T. Isolated C-terminal tail of FGF23 alleviates hypophosphatemia by inhibiting FGF23-FGFR-Klotho complex formation. PNAS. (2010) 107:407–12. 10.1073/pnas.0902006107 19966287 PMC2806769

[91] Tsai W-C Wu H-Y Peng Y-S Hsu S-P Chiu Y-L Chen H-Y. Effects of lower versus higher phosphate diets on fibroblast growth factor-23 levels in patients with chronic kidney disease: a systematic review and meta-analysis. Nephrol Dial Transplant. (2018) 33:1977–83. 10.1093/ndt/gfy005 29420827

[92] Bouma-de Krijger A Vervloet MG. Fibroblast growth factor 23: are we ready to use it in clinical practice? J Nephrol. (2020) 33:509–27. 10.1007/s40620-020-00715-2 32130720 PMC7220896

[93] Smith ER McMahon LP Holt SG. Method-specific differences in plasma fibroblast growth factor 23 measurement using four commercial ELISAs. Clin Chem Lab Med. (2013) 51:1971–81. 10.1515/cclm-2013-0208 23729624

[94] Di Marco GS Reuter S Kentrup D Grabner A Amaral AP Fobker M. Treatment of established left ventricular hypertrophy with fibroblast growth factor receptor blockade in an animal model of CKD. Nephrol Dial Transplant. (2014) 29:2028–35. 10.1093/ndt/gfu190 24875663 PMC4425841

[95] Andrukhova O Slavic S Smorodchenko A Zeitz U Shalhoub V Lanske B. FGF23 regulates renal sodium handling and blood pressure. EMBO Mol Med. (2014) 6:744–59. 10.1002/emmm.201303716 24797667 PMC4203353

[96] Czaya B Seeherunvong W Singh S Yanucil C Ruiz P Quiroz Y. Cardioprotective effects of paricalcitol alone and in combination with FGF23 receptor inhibition in chronic renal failure: experimental and clinical studies. Am J Hypertens. (2019) 32:34–44. 10.1093/ajh/hpy154 30329020 PMC6284753

[97] Freundlich M Li YC Quiroz Y Bravo Y Seeherunvong W Faul C. Paricalcitol downregulates myocardial renin-angiotensin and fibroblast growth factor expression and attenuates cardiac hypertrophy in uremic rats. Am J Hypertens. (2014) 27:720–6. 10.1093/ajh/hpt177 24072555 PMC3978945

[98] Leifheit-Nestler M Kirchhoff F Nespor J Richter B Soetje B Klintschar M. Fibroblast growth factor 23 is induced by an activated renin-angiotensin-aldosterone system in cardiac myocytes and promotes the pro-fibrotic crosstalk between cardiac myocytes and fibroblasts. Nephrol Dial Transplant. (2018) 33:1722–34. 10.1093/ndt/gfy006 29425341

[99] Matsui I Oka T Kusunoki Y Mori D Hashimoto N Matsumoto A. Cardiac hypertrophy elevates serum levels of fibroblast growth factor 23. Kidney Int. (2018) 94:60–71. 10.1016/j.kint.2018.02.018 29751971

[100] Richter M Lautze H-J Walther T Braun T Kostin S Kubin T. The failing heart is a major source of circulating FGF23 via oncostatin M receptor activation. J Heart Lung Transpl. (2015) 34:1211–4. 10.1016/j.healun.2015.06.007 26267742

[101] Andrukhova O Slavic S Odörfer KI Erben RG. Experimental myocardial infarction upregulates circulating fibroblast growth factor-23. J Bone Miner Res. (2015) 30:1831–9. 10.1002/jbmr.2527 25858796 PMC4973700

[102] Leifheit-Nestler M Grabner A Hermann L Richter B Schmitz K Fischer D-C. Vitamin D treatment attenuates cardiac FGF23/FGFR4 signaling and hypertrophy in uremic rats. Nephrol Dial Transplant. (2017) 32:1493–503. 10.1093/ndt/gfw454 28339837 PMC5837317

[103] Takashi Y Kinoshita Y Hori M Ito N Taguchi M Fukumoto S. Patients with FGF23-related hypophosphatemic rickets/osteomalacia do not present with left ventricular hypertrophy. Endocr Res. (2017) 42:132–7. 10.1080/07435800.2016.1242604 27754732

[104] Liu ES Thoonen R Petit E Yu B Buys ES Scherrer-Crosbie M. Increased circulating FGF23 does not lead to cardiac hypertrophy in the male hyp mouse model of XLH. Endocrinology. (2018) 159:2165–72. 10.1210/en.2018-00174 29635291 PMC5915960

[105] Pastor-Arroyo E-M Gehring N Krudewig C Costantino S Bettoni C Knöpfel T. The elevation of circulating fibroblast growth factor 23 without kidney disease does not increase cardiovascular disease risk. Kidney Int. (2018) 94:49–59. 10.1016/j.kint.2018.02.017 29735309

[106] Clinkenbeard EL Noonan ML Thomas JC Ni P Hum JM Aref M. Increased FGF23 protects against detrimental cardio-renal consequences during elevated blood phosphate in CKD. JCI Insight. (2019) 4:e123817. 10.1172/jci.insight.123817 30830862 PMC6478421

[107] Kuga K Kusakari Y Uesugi K Semba K Urashima T Akaike T. Fibrosis growth factor 23 is a promoting factor for cardiac fibrosis in the presence of transforming growth factor-β1. PLoS ONE. (2020) 15:e0231905. 10.1371/journal.pone.0231905 32315372 PMC7173860

[108] Roy C Lejeune S Slimani A Meester C de Ahn AS SA Rousseau MF. Fibroblast growth factor 23: a biomarker of fibrosis and prognosis in heart failure with preserved ejection fraction. ESC Heart Fail. (2020) 7:2494–507. 10.1002/ehf2.12816 32578967 PMC7524237

[109] Shalhoub V Shatzen EM Ward SC Davis J Stevens J Bi V. FGF23 neutralization improves chronic kidney disease-associated hyperparathyroidism yet increases mortality. J Clin Invest. (2012) 122:2543–53. 10.1172/JCI61405 22728934 PMC3386816

[110] Burnett S-AM Gunawardene SC Bringhurst FR Jüppner H Lee H Finkelstein JS. Regulation of C-terminal and intact FGF-23 by dietary phosphate in men and women. J Bone Miner Res. (2006) 21:1187–96. 10.1359/jbmr.060507 16869716

[111] Isakova T Barchi-Chung A Enfield G Smith K Vargas G Houston J. Effects of dietary phosphate restriction and phosphate binders on FGF23 levels in CKD. CJASN. (2013) 8:1009–18. 10.2215/CJN.09250912 23471131 PMC3675851

[112] Goto S Nakai K Kono K Yonekura Y Ito J Fujii H. Dietary phosphorus restriction by a standard low-protein diet decreased serum fibroblast growth factor 23 levels in patients with early and advanced stage chronic kidney disease. Clin Exp Nephrol. (2014) 18:925–31. 10.1007/s10157-014-0947-4 24578219

[113] Larsson T Nisbeth U Ljunggren O Jüppner H Jonsson KB. Circulating concentration of FGF-23 increases as renal function declines in patients with chronic kidney disease, but does not change in response to variation in phosphate intake in healthy volunteers. Kidney Int. (2003) 64:2272–9. 10.1046/j.1523-1755.2003.00328.x 14633152

[114] Vervloet MG van Ittersum FJ Büttler RM Heijboer AC Blankenstein MA Wee PM ter. Effects of dietary phosphate and calcium intake on fibroblast growth factor-23. CJASN. (2011) 6:383–9. 10.2215/CJN.04730510 21030580 PMC3052230

[115] Ix JH Isakova T Larive B Raphael KL Raj DS Cheung AK. Effects of nicotinamide and lanthanum carbonate on serum phosphate and fibroblast growth factor-23 in CKD: the COMBINE trial. JASN. (2019) 30:1096–108. 10.1681/ASN.2018101058 31085679 PMC6551774

[116] Chang Y-M Tsai S-C Shiao C-C Liou H-H Yang C-L Tung N-Y. Effects of lanthanum carbonate and calcium carbonate on fibroblast growth factor 23 and hepcidin levels in chronic hemodialysis patients. Clin Exp Nephrol. (2017) 21:908–16. 10.1007/s10157-016-1362-9 27928636

[117] Zhang C Wang S Zhao S Zhang X. Effect of lanthanum carbonate on coronary artery calcification and bone mineral density in maintenance hemodialysis patients with diabetes complicated with adynamic bone disease: a prospective pilot study. Medicine. (2017) 96:e8664. 10.1097/MD.0000000000008664 29137107 PMC5690800

[118] Vlassara H Uribarri J Cai W Goodman S Pyzik R Post J. Effects of sevelamer on HbA1c, inflammation, and advanced glycation end products in diabetic kidney disease. CJASN. (2012) 7:934–42. 10.2215/CJN.12891211 22461535 PMC3362316

[119] Moe SM Chertow GM Parfrey PS Kubo Y Block GA Correa-Rotter R. Cinacalcet, fibroblast growth factor-23, and cardiovascular disease in hemodialysis: the Evaluation of Cinacalcet HCl Therapy to Lower Cardiovascular Events (EVOLVE) Trial. Circulation. (2015) 132:27–39. 10.1161/CIRCULATIONAHA.114.013876 26059012

[120] Schön A Leifheit-Nestler M Deppe J Fischer D-C Bayazit AK Obrycki L. Active vitamin D is cardioprotective in experimental uraemia but not in children with CKD Stages 3-5. Nephrol Dial Transplant. (2021) 36:442–51. 10.1093/ndt/gfaa227 33241290

[121] Suassuna PG Cherem PM de Castro BB Maquigussa E Cenedeze MA Lovisi JC. αKlotho attenuates cardiac hypertrophy and increases myocardial fibroblast growth factor 21 expression in uremic rats. Exp Biol Med. (2020) 245:66–78. 10.1177/1535370219894302 31847589 PMC6987748

[122] Sinha MD Turner C Goldsmith DJ. FGF23 concentrations measured using intact assays similar but not interchangeable. Int Urol Nephrol. (2013) 45:1821–3. 10.1007/s11255-013-0451-x 23649347

[123] Dirks NF Smith ER van Schoor NM Vervloet MG Ackermans MT Jonge R de. Pre-analytical stability of FGF23 with the contemporary immunoassays. Clin Chim Acta. (2019) 493:104–6. 10.1016/j.cca.2019.02.032 30826370

[124] Smith ER Ford ML Tomlinson LA McMahon LP Rajkumar C Holt SG. FGF23 adds value to risk prediction in patients with chronic kidney disease. Bone. (2012) 51:830–1; author reply 832–3. 10.1016/j.bone.2012.05.017 22683387

[125] Edmonston D Wojdyla D Mehta R Cai X Lora C Cohen D. Single measurements of carboxy-terminal fibroblast growth factor 23 and clinical risk prediction of adverse outcomes in CKD. Am J Kidney Dis. (2019) 74:771–81. 10.1053/j.ajkd.2019.05.026 31445926 PMC6875624

[126] Artunc F Nowak A Müller C Peter A Heyne N Häring H-U. Mortality prediction using modern peptide biomarkers in hemodialysis patients—a comparative analysis. Kidney Blood Press Res. (2014) 39:563–72. 10.1159/000368468 25531828

[127] Nowak N Skupien J Smiles AM Yamanouchi M Niewczas MA Galecki AT. Markers of early progressive renal decline in type 2 diabetes suggest different implications for etiological studies and prognostic tests development. Kidney Int. (2018) 93:1198–206. 10.1016/j.kint.2017.11.024 29398132 PMC5911430

[128] Alderson HV Ritchie JP Middleton R Larsson A Larsson TE Kalra PA. FGF-23 and Osteoprotegerin but not Fetuin-A are associated with death and enhance risk prediction in non-dialysis chronic kidney disease stages 3-5. Nephrology. (2016) 21:566–73. 10.1111/nep.12664 27334353

[129] Emrich IE Brandenburg V Sellier AB Schauerte J Wiedenroth J Untersteller K. Strength of fibroblast growth factor 23 as a cardiovascular risk predictor in chronic kidney disease weaken by ProBNP adjustment. Am J Nephrol. (2019) 49:203–11. 10.1159/000497125 30808827

[130] Udell JA Morrow DA Jarolim P Sloan S Hoffman EB O'Donnell TF. Fibroblast growth factor-23, cardiovascular prognosis, and benefit of angiotensin-converting enzyme inhibition in stable ischemic heart disease. J Am Coll Cardiol. (2014) 63:2421–8. 10.1016/j.jacc.2014.03.026 24727254 PMC4213068

